# Platelet-Rich Plasma in Treatment of Temporomandibular Joint Dysfunctions: Narrative Review

**DOI:** 10.3390/ijms20020277

**Published:** 2019-01-11

**Authors:** Francesca Zotti, Massimo Albanese, Luigi Fabrizio Rodella, Pier Francesco Nocini

**Affiliations:** 1 Department of Surgery, Dentistry, Paediatric and Gynaecology, University of Verona. Policlinico G. B. Rossi. Piazzale L. Scuro n.10, 37134 Verona, Italy; massimo.albanese@univr.it (M.A.); pierfrancesco.nocini@univr.it (P.F.N.); 2 Section of Anatomy and Pathophysiology, Department of Clinical and Experimental Sciences, University of Brescia, 25100 Brescia, Italy; luigi.rodella@unibs.it

**Keywords:** temporomandibular joint (TMJ), temporomandibular joint disorders (TMD), platelet-rich plasma, arthrocentesis, injection, hyaluronic acid

## Abstract

Background: The aims of this narrative review were to examine up-to-date literature in order to evaluate the effectiveness of arthrocentesis or injections with platelet-rich plasma in temporomandibular affections and to compare them to arthrocentesis alone or with hyaluronic acid (HA) or to hyaluronic acid injections. Methods: The search of international literature was made on the PMC, PubMed and Cochrane databases, including all full-length text of studies on humans focused on osteoarthritis and disc displacements and their treatment with platelet-rich plasma arthrocentesis or injections. All design studies were included in the review and they were examined for three different outcomes: pain, joint sound and mandibular motion. English papers were only selected. Results: Even though the low number of studies in this field, arthrocentesis with platelet-rich plasma and platelet-rich plasma injections in temporomandibular disorders’ management were found to be effective in reducing pain and joint sound as well as in improving mandibular motion in a maximum follow-up of 24 months. Conclusion: Comparison to arthrocentesis alone or to HA use in arthrocentesis or by injections provided encouraging results in terms of the effectiveness of platelet-rich plasma use.

## 1. Introduction

Temporomandibular joint disorders (TMDs) affect the jaw joints and related structures causing internal derangement of joint space, bone alterations and degenerative pathologies. Frequent signs and symptoms of TMDs are pain, joint noise, limited range of motion, impaired jaw function, deviation or deflection upon mouth opening and closing or open locking [1,2].

Internal derangements of temporomandibular joint (TMJ) include disc displacements, with or without reduction, often responsible for joint sounds, pain and discomfort in the TMJ area. Generally, joint displacements are strictly related to the structure and cinematics of the TMJ and masticatory system [3], however they can be also caused by peculiar anatomical morphology of the condyle, glenoid fossa and articular eminence [4]. Furthermore, age, dentition and patterns of masticatory muscle could be important factors in determining or maintaining temporomandibular joint dislocations [5]. Disc displacements occur when a disc is located outside of its normal position in the joint spaces. The normal disc position means a 12 o’clock position of the posterior band and a 10 o’clock position of the intermediate zone of the disc. The disc has a “bow tie” shape with a thin intermediate zone with the narrowest inter-bony distance. Displacement of the disc can present displacements in any direction, but anterior disc displacement is most common. In dislocation with reduction, the posterior band of the disc was located anteriorly to the condylar head in the closed position, but with a normal disc–condyle relationship with the mouth opened by 1 inch. In non-reducing displacement, the posterior band was positioned anteriorly to the condyle both with the mouth closed and opened 1 inch [6].

Disc displacement with or without reduction of the TMJ is an intracapsular dysfunction that leads to degenerative changes in the disc and articular surface [7].

Osteoarthritis (OA) of the TMJ is characterized by degenerative alterations of bone, cartilage and supporting tissues causing pain, stiffness and loss of function [8]. It is more common in the female than the male and it has a degenerative pattern leading to bone erosion, sclerosis and bony alterations such as osteophytes and flattening of condyle surface [9]. In most of cases, limitations in motion occurs and patients are not able to effectively chew or achieve a normal mouth opening without pain.

Different treatments of OA and disc displacement are reported in literature and performed in clinical practice and they include conservative therapy such as painkillers, resting the jaw, splints and physiotherapy and surgical interventions as arthrocentesis, disc repositioning or discectomy in non-responder patients.

Treatment outcomes of arthrocentesis of the TMJ are improvements of mouth opening and pain relief during the mandibular motion with consequent enhancing of it [8,10].

Even though literature reports the possibility to perform solely arthrocentesis to reach these outcomes [8,11], further studies have been carried out to better investigate the effectiveness of different techniques to perform arthrocentesis by using growing factors, autologous solutions or alternative materials; the materials most used are platelet-rich plasma (PRP) and hyaluronic acid (HA) in arthrocentesis or by injection.

Platelet-rich plasma is produced by centrifuging heparinized whole autologous blood for 15 min and separating the platelets from the other blood components. Later, the platelets are diluted with normal saline solution to obtain the optimal concentration. This represents an emerging regenerative therapy for injuries in the orthopaedic field with encouraging results showing anti-inflammatory, analgesic and antibacterial properties [12,13,14].

PRP has recently been considered as an orthobiological adjuvant treatment. It also restores intra-articular hyaluronic acid, increases glycosaminoglycan chondrocyte synthesis, balances joint angiogenesis, and provides a scaffold for stem cells migration. Basic scientific studies have indicated that PRP stimulates cell proliferation and the production of cartilage matrix by chondrocytes and bone marrow-derived mesenchymal stromal cells and increases the production of hyaluronic acid by synoviocytes [15].

HA is a high-molecular-weight glycosaminoglican naturally present in synovial fluid and participating in joint lubrification. Injections of HA have been widely used in the treatment of TMDs in single-dose or repeated or associated with other procedures, such as arthrocentesis or arthroscopy, and several published studies show positive and encouraging results in improvement of mouth opening and pain relief [1,16,17,18].

Despite the extensive literature on the use of PRP in the treatment of articular disorders in orthopedics, its application in TMDs is quite unexplored, and therefore this narrative review should deeper investigate PRP effectiveness in TMDs treatment.

The primary aim of this review is to assess the effectiveness of PRP in arthrocentesis or by injections in terms of reducing pain, joint sounds and improving mandibular motion in patients affected by osteoarthritis or internal derangement of the TMJ.

The secondary aim is to compare the effectiveness of PRP in arthrocentesis to HA in arthrocentesis or injected to arthrocentesis alone in affected patients.

## 2. Research Methods

The methodological approach of this work is a narrative review in order to summarize the main findings of literature, to better outline and to improve knowledge in the field of interest [19]. Specifically, we used concepts proposed by Egger et al. to perform narrative review of the literature according to the following steps (Table 1) [20].

Ten papers included were subsequently divided into different subgroups of interest based on our outcomes and study variables in order to investigate in depth the particular contribution of each article. We chose specific keywords for each topic in order to highlight our outcomes in the papers (Table 3).

First, in order to examine signs and symptoms of TMJ OA and internal derangements, we based our evaluation on all papers that reported an evaluation and outcome. Considering that VAS scale, ability in mouth opening and joint sound have been used as parameters in all studies, we considered them as rating parameters. Second, to investigate the treatment proposed, we focused attention on all works proposing PRP arthrocentesis or PRP injection or arthrocentesis in TMJ. Conservative therapies in OA and internal derangements, such as painkillers or dental splints, were not taken into account because not objective of this review, except where combined with arthrocentesis or PRP injections.

## 3. Results

**Figure 1:**
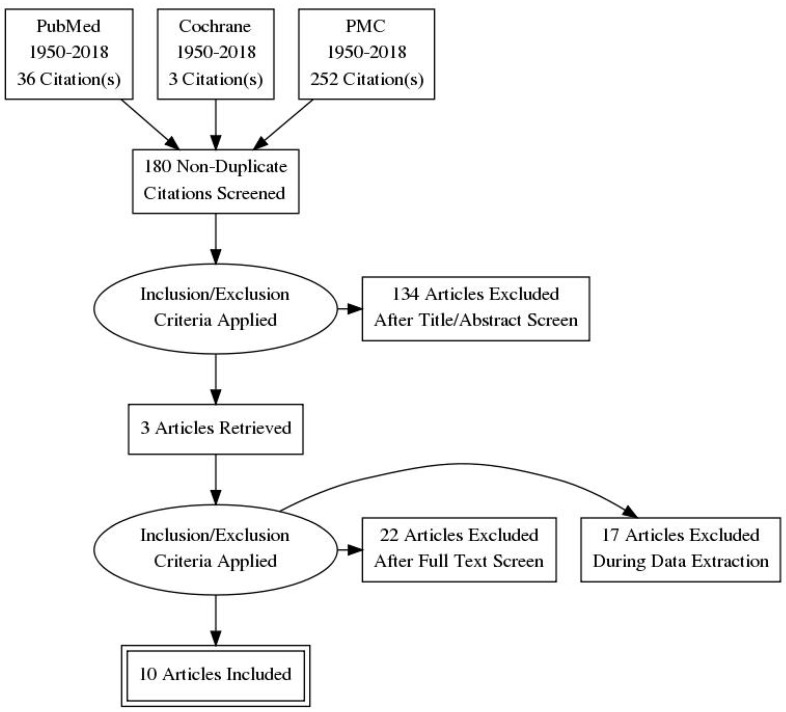
Literature selection process.

Literature screening led to the evaluation of 10 full-length articles (Figure 1).

Different kinds of studies were taken into consideration, such as randomized controlled trials (RCT), randomized prospective studies, observational studies, clinical studies and retrospective cohort studies.

Table below shows characteristics of studies itemised by intervention, sample size, studies’ design and outcomes measurement (Table 4).

In four studies (Hegab et al., Kiliç et al., 2015 and 2016 and Lin et al.) out of 10 osteoarthritis was the only affect tested; however in one work [21] (Giacomello et al.), authors evaluated also anterior disc displacement without reduction. Fernandez-Ferro evaluated both osteoarthritis an disc displacement with or without reduction [22]. Hanci et al. recruited a sample suffering from disc displacement with reduction [10], Pihut and al. evaluated a sample affected by general temporomandibular disfunctions [23], and Yang et al. and Al-Delayme et al. evaluated non-reducing disc displacement samples [2,24].

One study out of 10 compared PRP injections to arthrocentesis [10] (Hanci et al.), two studies out of 10 compared PRP injections to HA injections [15,22] (Hegab et al., Frenandez-Ferro et al.). Three studies tested different doses of PRP injections without a control group (Giacomello et al., Pihut et al., Al-Delayme et al.,) and Yang et al. tested LPCGF (Liquid Phase Concentrated Growth Factor) injection combined with centric relation occlusal splint without comparison [2,21,23,24]. In both studies of Kiliç et al. (2015, 2016) PRP and arthrocentesis and PRP injection was compared with arthrocentesis alone (2015) and with HA arthocentesis [8,9]. In Lin et al. PRP arthrocentesis was compared with PRP injection [25].

### 3.1. Outcome of Pain Improvement

Pain was measured by VAS (visual analogue scale) in all works evaluated. Statistically significant results in terms of pain improvement were highlighted in all works examined, except in Lin et al. [25].

Intra-group and inter-groups differences were noticed in all works where a control group was involved. In those studies where control groups were not present, statistical differences were noted between the baseline and the end of follow-up in the study group.

In Lin’s work, arthrocentesis plus PRP was compared to PRP injection in osteoarthritis and the two groups did not show statistically significant differences in TMJ arthralgia [25]. Results of VAS scores from all studies were found to be similar in values, except in Fernandez-Ferro et al. where a slighter improvement was noticed, probably due to the larger sample tested [22].

Pain seems to improve when PRP is used, both by injections combined with arthrocentesis. Furthermore, PRP injections were found to be more effective than HA injections (Hegab et al., Fernandez-Ferro et al.) [15,22].

### 3.2. Outcome of Joint Sound

In two studies joint sound was not evaluated (Giacomello et al. and Fernandez-Ferro et al.) [21,22]. In three studies the joint sound was evaluated using VAS scale (Kiliç et al. 2015, 2016 and Al-Delayme) [2,8,9], in three other studies it was calculated on joints affected by sound or crepitus (Hanci et al., Hegab et al., Yang et al.) [10,15,24], and in another two number of patients reporting sound was scored (Pihut et al., Lin et al.) [23,25].

In all of the works analysed joint sound was found to improve during follow up. In Hegab et al. and in Pihut et al. results were not statistically significant, nevertheless an improvement was noticed [15,23]. In particular, Hegab et al. reported improvements of joint sound when treated with PRP injection compared to HA injection at 1 month of follow-up, however this improvement became equal at 12 months follow-up [15].

In both studies carried out by Kiliç et al. statistically significant inter-groups resulting in decreasing of joint sound were reported [8,9]. Hanci et al. showed also statistical differences between two compared groups, as well as in Yang et al. and in Al-Delayme where statistical differences in outcome were reported comparing baseline to end of follow-up [2,10,24].

In Lin et al. at 1 month and at 12 months statistically significant improvement of joint crepitus sound was detected in patients treated by arthrocentesis and PRP. However, authors reported no statistical differences until 12 months between two groups (arthrocentesis and PRP compared to PRP injections) demonstrating a similar improvement of joint crepitus sound in both groups [25].

### 3.3. Mandibular Motion Outcome

Different definitions of this outcome were reported by authors, for example mandibular opening, mandibular motion, mouth voluntary opening and maximum mouth opening, minimal interincisal opening, range motion. We generally assumed all of these as mandibular motion.

In all studies, except Yang et al., mandibular motion was considered and tested at baseline and at the end of follow-up; furthermore, comparison between groups was carried out where a control group was present [24].

In Giacomello et al. (2 PRP Injections) differences in mandibular opening between pre-injection and post-injection were statistically significant [21].

Hanci et al. (PRP injection vs. arthrocentesis) investigated minimal interincisal opening founding no statistically significant differences between study and control group [10].

Pihut et al. (PRP injection) reported a decrease of mandibular motion but the results are not clearly explained [23].

Results in mandibular voluntary opening in Hegab et al. (PRP injections vs. HA injections) were found to be statistically different between two groups [15]. In Kiliç et al. (2015) (PRP arthrocentesis vs. arthrocentesis) results in maximum mandibular opening were not statistically different in two groups whereas in the work of 2016 (PRP arthrocentesis vs. HA arthrocentesis) statistical differences were noticed between study and control group [8,9]. Fernandez- Ferro et al. (PRP injection vs. HA injection) did not find differences with statistical relevance between the two groups in testing mouth opening before intervention and at the end of follow-up [22].

In Al-Delayme et al. (PRP injections), statistical differences between baseline and end of follow-up were noticed [2].

Lin et al. (PRP arthrocentesis vs. PRP injections) showed a statistical significant improvement in both groups after interventions (range motion higher than 6 mm), however no differences were noticed in terms of mouth-assisted opening [25].

Yang et al. did not evaluate range motion outcome [24].

### 3.4. Risk of Bias Assessment

Selected studies differ from each other due to heterogeneity of sample size and TMJ affections tested. The mean age of patients is ranged between 25.4 and 47.64 years.

Only four studies were RCT (Hanci et al., Hegab et al., Kiliç et al 2015 and 2016) [8,9,10,15] and one was randomized prospective study (Fernandez-Ferro et al.) [22]. In three studies allocation concealment and randomization technique were clearly explained (Kiliç et al. 2016, Fernandez-Ferro et al. and Hegab et al.) [8,15,22].

Three studies did not have control group for comparisons (Giacomello et al., Pihut et al., Al-Delayme et al.) [2,21,23].

Two studies lacked outcomes on joint sound (Giacomello et al., Fernandez-Ferro et al.) [21,22].

All studies were considered at low risk of bias for selective reporting.

## 4. Discussion

The weak point of this work is lack of studies and their heterogeneity. Risk of bias assessment clearly shows this weakness; however, the core aim of this review was to assess the effectiveness of PRP use in TMJ affections, thus we focused our attention on all studies responding to this topic. Literature does not provide a large spectrum of studies to perform a systematic review with subsequent meta-analysis.

Because of limited literature, an open search strategy was performed in order to include a larger number of studies. This methodological decision, however, led to difficulties in evaluating samples’ baseline conditions and outcomes.

The first significant limitation in this work was the diagnostic criterium used in each study, some study included OA patients, others considered also patients suffering from anterior disc displacement with or without reduction. This meant heterogeneity in sample evaluated and subsequent difficulties in comparing results of effectiveness of different therapeutic strategies. A further restriction we ran into were the different ways to perform TMDs diagnosis in each study. Some studies used clinical diagnosis, others magnetic resonance imaging (MRI) and others CBCT. Studies that performed only a clinical evaluation might be considered critical in diagnosis strategy as instrumental assessment are recommended in literature [26].

A further crucial point to take into account in evaluating bias are different ways used to prepare PRP solutions in the studies analysed. Fernandez-Ferro et al. and Kiliç et al. (2015 and 2016) followed the protocol proposed by Anitua et al. [27]. Lin et al. and Yang et al. followed Saccos’s protocol for PRP preparation and management [28]. Giacomello et al. proposed to use PRGF-Endoret (plasma rich in growth factors) with CaCl added and Al-Delayme et al. also added CaCl to PRP solution extracted [29]. Other authors proposed their own protocols for preparation, centrifugation and extraction of PRP as well as times of administration. These differences did not allow for making considerations based on standardised procedures and, therefore, the outcomes’ evaluation might be influenced. Moreover the characterization of PRP contents in each solution used in different studies analysed represented a critical point when we made comparisons in the effectiveness of PRP injections and PRP combined with arthrocentesis. Patients’ characteristics from which PRP was extracted and its preparation were not well specified in studies and literature that shows differences in effectiveness of PRP based on blood properties and contents [30]. For example concentration of specific growth factors, or cytokines could affect the PRP properties and it is an aspect to evaluate when comparing studies [31].

A further point is the injection technique of PRP; each author described his own surgical approach in administration of PRP. This aspect could be an additional critical issue in this review, even though, in our opinion, this does not represent a great matter of concern because PRP solution reached the intracapsular area in all cases.

In some studies, pain outcome was reported as TMJ arthralgia and other authors referred to myofascial pain; we assumed both conditions as pain outcome in order to evaluate changes after different therapies. The same approach was taken in evaluating mandibular motion because of differences within outcomes in studies. We considered as mandibular motion all outcomes proposed, such as mandibular opening (Giacomello et al.) [21], minimal interincisal opening (Hanci et al.) [10], mandibular motion (Pihut et al.) [23], mouth voluntary opening (Hegab et al.) [15], maximum mouth opening (Al-Delayme et al., Kiliç et al. 2015, 2016) [2,8,9], mouth opening (Fernandez-Ferro et al.) [22] and range motion and mouth-assisted opening (Lin et al.) [25]. With regards to interventions, we considered both injections of PRP and arthrocentesis with PRP not taking into account different methods to inject PRP or to perform arthrocentesis due to a shortage of studies available. Published data about PRP treatment for TMDs with which to establish application protocols are limited; however, authors did not report complications in using different techniques or composition of solutions [32,33].

Follow-up duration varied among studies and this aspect made it impossible to directly compare them.

Taking into account these limitations, based on recent literature findings and considerations about PRP use and TMDs’ etiopathogenetic behaviours, comparisons and evaluations of outcomes among all studies were performed, albeit at the expense of the methodological rigour.

In recent years, literature demonstrated that excessive metabolic reactions occur in TMDs conditions and, therefore, changes in microenvironment around cartilage and bone with subsequent damage appear [34]. Consequently pain and dysfunction in mandibular motion are related to an increase of pressure in the joint and to the great amount of cytokines in synovial liquid [22]. Literature suggests that PRP injection could improve these conditions, not only thanks to the expansion of the joint cavity, but also because of growth factors which can restore disc, capsule and retrodiscal pad and inhibit pro-inflammatory cytokines [35,36,37]. According to these findings, results of this review show clear improvement when PRP injections were performed, regardless of injection modalities or periodicity. This occurs even when PRP injections are compared with HA injections (Hegab et al.). In particular, Hegab et al. show HA injections are comparable in effects to PRP injections in a medium follow-up; however, PRP allows better performances at the long-term follow-up without recurrence of pain and joint sound at 12 months [15]. Al-Delayme reported the effectiveness of PRP injection as primary treatment of non-reducing disk displacement, however assumed this to be an optimal duration of therapy 6 months, after which it might be considerable an additional injection [2]. Results of efficacy and modes of action of PRP use in TMDs are rare, and hence knowledge resulting from orthopaedic research: the mechanism for leading the patients to better condition with PRP injection remains obscure, nevertheless most authors report that PRP is a natural source of autologous growth factors and it could improve cartilage repairs [38,39]. Authors tested PRP and HA in the treatment of knee joint osteoarthritis and results were better in the group of patients who received PRP injections after a 3 and 6 months follow-up [40].

The inflammatory modulating capability of PRP was found to be crucial in reducing signs and symptoms of TMDs and this aspect seemed to play a key role in results of this review. In all studies where PRP was used, improvement of outcomes was detected. However debated results are presented in Kiliç (2016) regarding the effectiveness of PRP compared to HA in arthrocentesis [8]. Both biological factors resulted in significant clinical improvements of all outcomes, and it can indeed be extrapolated that PRP is perhaps inferior to HA, because results from four additional PRP injections seemed to be not better than one session of HA injection. There are no strong scientific data about temporomandibular joints to accept or reject this assumption, and other joints were more deeply investigated in orthopaedic field, despite with controversial results. Raeissadat et al. compared the efficacy of two PRP injections and three HA injections for treatment of knee osteoarthritis and the results indicated that two PRP injections were more efficacious than three HA injections in reducing knee symptoms [41]. With respect to TMDs, Fernandez-Ferro et al. reported how the infiltration of PRGF after an arthroscopy procedure shows better results for both pain and mouth opening with respect to HA. However no statistical difference was reported in the mouth opening outcome.

In view of this, it is difficult to gain results of PRP and HA comparisons, and there is no strong evidence in literature. It should certainly be considered that HA is more readily available, it does not require any invasive preparation procedure involving patients, and it is characterized by good tolerability and therapeutic effects (pain relief) [42,43]. Therefore stronger evidence might be required in order to chart the best course of treatment, also taking into account the invasiveness of preparation procedures and patients needing. Based on results of this narrative review, where pain relief is needed, PRP injections should be preferred to HA injections. The same therapeutic strategy could be undertaken when mandibular motion improvement is required, even if, at 18 months follow-up, results about this outcome seem to be overlapping both with PRP and HA injections in the case of OA and disc displacements (Fernandez-Ferro et al.) [22]. Furthermore, where joint sound improvement is needed, PRP seems to be preferable to HA (Hegab et al.) [15]; however, also with reference to this outcome, the results become the same at the end of the follow-up. However, it might be considered whether joint sound improvement is a primary outcome desired or it is secondary to pain relief and mandibular motion. Patients actually first require a decrease in pain and enhancement of mandibular cinematics, and therefore joint sound improvement.

In light of these findings and taking into account the limited and heterogeneous studies in literature, we might assume that the best therapeutic choice among PRP and HA should be driven by a patient’s needs, their systemic conditions, and the outcomes to be reached. Until further studies and extended follow-ups are undertaken, it could be advisable to prefer PRP arthrocentesis or injections; however, sometimes it can be difficult to obtain PRP and, therefore, HA could be the best choice.

Comparing PRP injections or PRP combined with arthrocentesis to arthrocentesis alone, the results were more encouraging and clear. Statistically significant values were highlighted in Kiliç et al. (2015) [9] where patients who underwent four PRP injections show greater masticatory efficiency than the patients without PRP injections (only arthrocentesis), and CBCT findings confirmed two-fold better reparative remodelling in OA [9]. An interesting consideration could be made about Lin et al., where PRP injections and PRP arthrocentesis were compared, showing reparative remodeling in both treatments. This study reported also that an arthrocentesis prior to one single PRP injection could lead to more satisfactory outcomes, where TMJ OA is accompanied by clinical symptoms [25].

In accordance with the literature, PRP was found to have relevant antinflammatory and regenerative properties, capability of modulating synovial cell biology, increasing HA concentration and stabilizing angiogenesis [44] and these peculiar features probably provided PRP with higher efficacy also in the treatment of TMDs compared to arthrocentesis and HA injections.

PRP use, by injection or combined with arthrocentesis, in the treatment of TMDs was found to be more effective than arthrocentesis alone or combined with HA. This could be attributable to the inflammatory modulating capability of PRP in TMJ. More studies, especially RCTs, are required to fix these results.

## Figures and Tables

**Table 1:** Methodological approach to review.

Step	General Activities	Specific Activities
I	Formation of working group	One maxillofacial surgeon expert in arthrocentesis of TMJ, as clinical and methodological operator
		One medical doctor expert in head and neck anathomy and PRP, as clinical and methodological operator
		One researcher expert in TMJ disorders, as methodological operator
II	Formulation of the review questions	Evaluation of the state of art inTMJ osteoarthritis and arthrocentesis. Analysis of main effects of osteoarthritis in TMJ and its treatment
III	Identification of relevant studies on PubMed, PMC, Cochrane	1. Identification of keywords in the field of interest 2. Use of Boolean operators (AND; OR; NOT) 3. Advanced search (Table 2 for serach strategy) 4. Inclusion criteria: papers published from 1950 to 2018; language: english; all types of full text articles 5. Elimination of duplicate 6. Manual search through the references in selected articles
IV	Anaysis and presentation	Data extrapolated from all revised studies were shown in tables in form of narrative review

Step I: formation of a working group composed of three members. Each of them performed as methodological reviewer and two of them also as clinical expert. Step II: Formulation of review questions derived from up-to-date knowledge on TMJ osteoarthritis, treatment strategies and outcomes. Crucial step to effectively build a search strategy. Step III: using keywords concerning the topic to perform search in databases (PubMed, PMC, Cochrane). Boolean operators were all applied to search in order to establish proper relationships among concepts (Table 2). Step IV: analysis and presentation of all extrapolated data were collocated in table in form of narrative review and explanation in the results section of this paper.

**Table 2:** Search strategy.

Search	Database
((“platelet-rich plasma”[MeSH Terms] OR (“platelet-rich”[All Fields] AND “plasma”[All Fields]) OR “platelet-rich plasma”[All Fields] OR (“platelet”[All Fields] AND “rich”[All Fields] AND “plasma”[All Fields]) OR “platelet rich plasma”[All Fields]) AND (“temporomandibular joint”[MeSH Terms] OR (“temporomandibular”[All Fields] AND “joint”[All Fields]) OR “temporomandibular joint”[All Fields] OR “tmj”[All Fields])) NOT (extraction[All Fields] AND (“tooth”[MeSH Terms] OR “tooth”[All Fields] OR “teeth”[All Fields]))	PMC
(((“joint diseases”[MeSH Terms] OR (“joint”[All Fields] AND “diseases”[All Fields]) OR “joint diseases”[All Fields] OR “arthrosis”[All Fields]) AND (“temporomandibular joint”[MeSH Terms] OR (“temporomandibular”[All Fields] AND “joint”[All Fields]) OR “temporomandibular joint”[All Fields] OR “tmj”[All Fields])) AND ((“arthritis”[MeSH Terms] OR “arthritis”[All Fields]) AND (“temporomandibular joint”[MeSH Terms] OR (“temporomandibular”[All Fields] AND “joint”[All Fields]) OR “temporomandibular joint”[All Fields] OR “tmj”[All Fields]))) AND (“platelet-rich plasma”[MeSH Terms] OR (“platelet-rich”[All Fields] AND “plasma”[All Fields]) OR “platelet-rich plasma”[All Fields] OR (“platelet”[All Fields] AND “rich”[All Fields] AND “plasma”[All Fields]) OR “platelet rich plasma”[All Fields])	PMC
((((“temporomandibular joint”[MeSH Terms] OR (“temporomandibular”[All Fields] AND “joint”[All Fields]) OR “temporomandibular joint”[All Fields]) AND (“osteoarthritis”[MeSH Terms] OR “osteoarthritis”[All Fields])) AND (“osteoarthritis”[MeSH Terms] OR “osteoarthritis”[All Fields] OR “osteoarthrosis”[All Fields])) AND (disk[All Fields] AND (“displacement (psychology)”[MeSH Terms] OR (“displacement”[All Fields] AND “(psychology)”[All Fields]) OR “displacement (psychology)”[All Fields] OR “displacement”[All Fields]))) AND (“platelet-rich plasma”[MeSH Terms] OR (“platelet-rich”[All Fields] AND “plasma”[All Fields]) OR “platelet-rich plasma”[All Fields] OR (“platelet”[All Fields] AND “rich”[All Fields] AND “plasma”[All Fields]) OR “platelet rich plasma”[All Fields])	PMC
(((“joint diseases”[MeSH Terms] OR (“joint”[All Fields] AND “diseases”[All Fields]) OR “joint diseases”[All Fields] OR “arthrosis”[All Fields]) AND (“temporomandibular joint”[MeSH Terms] OR (“temporomandibular”[All Fields] AND “joint”[All Fields]) OR “temporomandibular joint”[All Fields] OR “tmj”[All Fields])) AND ((“arthritis”[MeSH Terms] OR “arthritis”[All Fields]) AND (“temporomandibular joint”[MeSH Terms] OR (“temporomandibular”[All Fields] AND “joint”[All Fields]) OR “temporomandibular joint”[All Fields] OR “tmj”[All Fields]))) AND (“platelet-rich plasma”[MeSH Terms] OR (“platelet-rich”[All Fields] AND “plasma”[All Fields]) OR “platelet-rich plasma”[All Fields] OR (“platelet”[All Fields] AND “rich”[All Fields] AND “plasma”[All Fields]) OR “platelet rich plasma”[All Fields])	PubMed
((Therapy/Broad[filter] AND ((“platelet-rich plasma”[MeSH Terms] OR (“platelet-rich”[All Fields] AND “plasma”[All Fields]) OR “platelet-rich plasma”[All Fields] OR (“platelet”[All Fields] AND “rich”[All Fields] AND “plasma”[All Fields]) OR “platelet rich plasma”[All Fields]) AND maxillofacial[All Fields])) NOT extraction[All Fields]) AND ((“osteonecrosis”[MeSH Terms] OR “osteonecrosis”[All Fields]) AND (“jaw”[MeSH Terms] OR “jaw”[All Fields]))	PubMed
(((“platelet-rich plasma”[MeSH Terms] OR (“platelet-rich”[All Fields] AND “plasma”[All Fields]) OR “platelet-rich plasma”[All Fields] OR (“platelet”[All Fields] AND “rich”[All Fields] AND “plasma”[All Fields]) OR “platelet rich plasma”[All Fields]) AND (“temporomandibular joint disorders”[MeSH Terms] OR (“temporomandibular”[All Fields] AND “joint”[All Fields] AND “disorders”[All Fields]) OR “temporomandibular joint disorders”[All Fields] OR (“tmj”[All Fields] AND “disorders”[All Fields]) OR “tmj disorders”[All Fields])) AND maxillofacial[All Fields]) NOT (extraction[All Fields] AND (“tooth”[MeSH Terms] OR “tooth”[All Fields] OR “teeth”[All Fields]))	PubMed
((“temporomandibular joint”[MeSH Terms] OR (“temporomandibular”[All Fields] AND “joint”[All Fields]) OR “temporomandibular joint”[All Fields] OR “tmj”[All Fields]) AND (“Pharmacol Res Perspect”[Journal] OR “prp”[All Fields])) AND (“surgery”[Subheading] OR “surgery”[All Fields] OR “surgical procedures, operative”[MeSH Terms] OR (“surgical”[All Fields] AND “procedures”[All Fields] AND “operative”[All Fields]) OR “operative surgical procedures”[All Fields] OR “surgery”[All Fields] OR “general surgery”[MeSH Terms] OR (“general”[All Fields] AND “surgery”[All Fields]) OR “general surgery”[All Fields])	PubMed
tmj, arthrocentesis, prp, osteoarthritis:ti, ab, kw (Word variations have been searched)	Cochrane

Inclusion criteria were: all types of articles, related only to humans, articles exclusively related to TMJ published form January 1950 to May 2018. Exclusion criteria were: articles for which full text was not available, were not in English, or were grey literature. Duplicate articles were eliminated and, from the articles retrieved in the first round of search, additional references were identified by a manual search among the cited references. Process of literature selection was reported following the PRISMA (Preferred Reporting Items for Systematic Reviews and Meta-analyses statement guidelines.

**Table 3:** Advanced and specific search.

Subgroups	Outcome	Topic	Specific Keywords Searched in Papers
1	Signs and symptoms improvement	Pain, Mandibular motion, Joint sounds	“Pain”; “Maximum mouth opening”; “VAS scale”; “Joint sound”; “Chewing”; “Stiff”, “Mandibular mobility”
2	Effectiveness of PRP-associated arthrocentesis	Arthrocentesis alone, Arthrocentesis hyaluronic acid (HA) associated, Arthrocentesis PRP associated, Arthrocentesis associated with other biomaterials or drugs	“Arthrocentesis”; “Platelet-rich-plasma”; “Hyaluronic acid”, “Corticosteroid”, “Growing factors”

**Table 4:** Results. SG: Sample Group; CG: Control Group; RDC/TMD: Research Diagnostic Criteria for Temporomandibular Disorders; DC/TMD: Research Diagnostic Criteria for Temporomandibular Disorders; CBCT: Cone Beam Computed Tomography.

Studies	Affection	Study Design	Diagnostic Criteria	Intervention	Sample	Endpoint	Outcomes	Results
Gicomello et al., 2014	Osteoarthritis, Non-reducing Anterior Displacement	Observational Study	Ortopantomography and magnetic resonance imaging (MRI)	2 PRP injections at 30 days	SG, N = 13, Mean age: 47.64	6 months	Pain (visual analogue scale, VAS); Mandibular opening (MMO)	Pain improvement from 7.69 ± 1.9 to 0.23 ± 0.63 (*). MMO improvement from 30.15 ± 4.44 to 39.54 ± 4.55 (*)
Hanci et al., 2014	Reducing Anterior Displacement	Randomized Controlled Trial (RCT)	MRI	SG: 1 singole PRP Injection, CG: Arthrocentesis	SG, N = 10, Mean age: 27.2. CG, N = 10, Mean age: 25.4	6 months	Pain (VAS); Minimal Interincisal Opening (MIO); Joint Sound (Number of Joints affected)	Pain improvement: SG from 6.69 ± 2.21 to 0.07 ± 0.27. CG: from 6.52 ± 2.29 to 2.76 ± 1.48 (*). MIO improvement: SG from 32 ± 8.53 to 39.7 ± 10.39. CG from 30.2 ± 9.41 to 36.3 ± 5.51 (**). Joint Sound improvement: SG: from 12 to 2. CG from 12 to 5 (*)
Pihut et al., 2014	Temporomandibular disfunctions	Clinical Study (Preliminary)	Clinical (RDC/TMD questionnaire)	PRP injection	SG, N = 10, Mean age: 37.6	6 weeks	Pain (VAS); Mandibular Motion (MM); Joint Sound (Number of patients)	Pain improvement: from 6.5 to 0.6 (*); MM decrease to 1 (not clear); Joint Sound: from 4 to 1
Hegab et al., 2015	Osteoarthritis	RCT	Radiography or MRI	SG: 3 PRP Injections, CG: 3 HA Injections	SG: N = 25, Mean age: 39. CG: N = 25, Mean age: 38.2	12 months	Pain (VAS); Mouth Voluntary Opening (MVMO); Joint Sound (Number of Joints affected)	Pain improvement: SG from 7.36 ± 1.14 to 0.4 ± 0.763. CG: from 6.96 ± 1.24 to 1.64 ± 1.35 (*). MVMO improvement: SG from 33.88 ± 3.08 to 41.56 ± 2.31. CG from 32.40 ± 2.72 to 39.28 ± 2.80 (*). Joint Sound improvement: SG > CG at 1 month, SG = CG at 12 months (**)
Al-Delayme et al., 2016	Non-reducing Anterior Displacement	Observational Study	Bilateral palpation and measurement of mouth opening	2 PRP injections	SG: N = 44, Mean age: 36.6	6 months	Pain (VAS); Maximum Mouth Opening (MMO); Joint Sound (VAS)	Pain improvement from 33.5 ± 22.4 to 18.3 ± 17.9 (*). MMO improvement from 26.4 ± 11.3 to 41.5 ± 8.65 (*). Joint Sound imprvement from 79.3 ± 12.8 to 2.9 ± 15 (*)
Kiliç et al., 2015	Osteoarthritis	RCT	Clinical (DC/TMDs) and CBCT	SG: Arthrocentesis + PRP 4 monthly PRP Injections, CG: Arthrocentesis	SG: N = 18, 32 Joints, Mean age: 32.22. CG: N = 12, 15 Joints, Mean age: 35.08	12 months	Pain (VAS); Maximum Mouth Opening (MMO) (VAS); Joint Sound (VAS)	Pain improvement: SG from 5.70 ± 1.35 to 1.02 ± 1.88. CG: from 6.83 ± 2.28 to 2.43 ± 4.08 (*). MMO improvement: SG: from 38.72 ± 7.84 to 38.39 ± 8.02 (**). Joint Sound: SG from 5.48 ± 3.46 to 0.70 ± 0.85. CG from 5.45 ± 3.27 to 0.75 ± 1.42 (*)
Kiliç et al., 2016	Osteoarthritis	RCT	Clinical (DC/TMDs) and CBCT	SG: Artrocentesis+PRP and 4 PRP Injections. CG: 1 singole Arthrocentesis + HA	SG: N = 18, 32 Joints, Mean age: 32.22. CG: N = 13, 17 Joints, Mean age: 28.08	12 months	Pain (VAS); Maximum Mouth Opening (MMO) (VAS); Joint Sound (VAS)	Pain improvement: SG from 5.70 ± 1.35 to 1.02 ± 1.88. CG: from 5.71 ± 2.54 to 0.54 ± 0.87 (*). MMO imrovement: SG from 38.72 ± 7.84 to 38.39 ± 8.02. CG from 44.23 ± 8.14 to 43.77 ± 6.39 (*). Joint Sound: SG from 5.48 ± 3.46 to 0.70 ± 0.85. CG from 5.81 ± 3.16 to 1.81 ± 3.04 (*).
Fernandez-Ferro et al., 2017	Osteoarthritis, Reducing or Non-reducing disc displacement	Randomized Prospective Study	MRI	SG: PRP Injection; CG: HA Injection	SG: N = 50, Mean age: 38.4. CG:N = 50, Mean age: 33.2	18 months	Pain (VAS); Mouth Opening (MO)	Pain improvement: SG from 8.35 ± 0.90 to 1.55 ± 1.90. CG from 8.14 ± 0.60 to 2.20 ± 1.43 (*). MO improvement: SG from 27.74 ± 4.65 to 37.23 ± 4.94. CG from 27.92 ± 5.08 to 36.54 ± 5.78 (**)
Yang et al., 2017	Non-reducing disc displacement	Retrospective Cohort Study	Clinical (DC/TMDs) and MRI	SG: LPCGF Injection + Centric Relation Occlusal Splint (Cros)	SG: N = 29, Mean age: 39.55	24 monts	Pain (VAS); Joint Sound (Number of joints affetcted)	Pain improvement from 4.72 ± 2.58 to 1.10 ± 1.72. Joint Sound improvement from 36 to 10 (*).
Lin et al., 2018	Osteoarthritis	Retrospective Study	Clinical (DC/TMDs) and CBCT	SG: Arthrocentesis + PRP: CG: PRP Injection	SG: N = 30, Mean age: 42.73; CG: N = 60, Mean age: 38.73	12 months	Pain (VAS); Range motion (>6 mm); Mouth Assisted Opening (MAO); Joint Crepitus Sound (Number of patients)	Pain improvement: SG = CG no improvement. Range Motion: SG from 47% to 0%, CG from 20% to 2% (*). MAO: SG and CG no improvementJoint crepitus sound: SG from 100% to 47% (**). SG vs. CG: differences not statistically significant.
